# The Year That Ebola Virus Took Over West Africa: Missed Opportunities for Prevention

**DOI:** 10.4269/ajtmh.14-0818

**Published:** 2015-02-04

**Authors:** Daniel G. Bausch

**Affiliations:** Tulane School of Public Health and Tropical Medicine, Department of Tropical Medicine, New Orleans, Louisiana; US Naval Medical Research Unit No. 6, Virology and Emerging Infections Department, Lima, Peru

I will always remember 2014 as the year that Ebola virus took over west Africa and thus, much of my time and life. The epicenters of the outbreak in Guinea and Sierra Leone are areas that I have worked in since 1996 on projects to build capacity to combat another viral hemorrhagic disease, Lassa fever, with the Centers for Disease Control and Prevention (CDC), World Health Organization (WHO), Tulane University, and others. I spent a lot time and made many friends and colleagues in the Forest Region of Guinea and the Kenema District of Sierra Leone, both at the heart of this Ebola outbreak. I have also responded to quite a few Ebola and Marburg virus outbreaks over the years. Therefore, it was natural that, when Ebola hit west Africa, I would get involved. Indeed, since April of 2014, it has pretty much been all Ebola all of the time, with me cycling constantly between my home in Lima, Peru and west Africa, Geneva, and Washington, consulting primarily with the WHO and the US Government.

It was in June in Kenema that the outbreak started to become increasingly personal; it seemed that almost every day another healthcare worker who was part of our Kenema Lassa (now Ebola) team who I had been working with for decades, some who I had even recruited, including the now sadly famous Dr. Sheik Humarr Khan,^1^ was coming down with a fever, testing positive for Ebola, and becoming a case rather than a caregiver. It was like some surrealistic nightmare—“I dreamt that an Ebola outbreak hit Kenema, and everyone I knew was getting infected and dying.” A few months later, the dream-like quality extended to the United States, with imported cases, occasional secondary transmission, and much public panic.

Pretty much every day since then, the persistent question, one that countless journalists have asked me, has been “how did this happen?” The public health corollary of the question, how I ask it to myself, is “how could this all have been prevented?” Journalists focus on the big picture and short term: what might governments and international organizations have done differently over the last 10 months? These are valid questions, but with such close ties to the region and people, I cannot help but personalize and deepen the question. What might have been done differently in my projects in the region over the past 10 years that could have helped avert this humanitarian disaster? I suppose that there is some arrogant-sounding “I told you so” in the answers, but the accusation is just as often directed at myself. Boiling it all down, I see at least five areas of missed opportunity that might have made a difference.

## Building and Maintaining Diagnostic Laboratory and Outbreak Response Capacity

**Figure 1.**
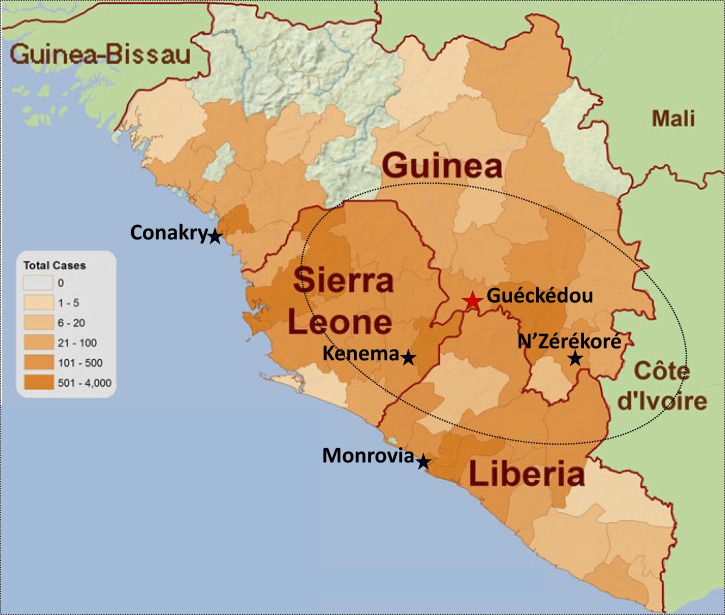
Map of the Mano River Union countries (Sierra Leone, Guinea, and Liberia) showing the total number of cases of Ebola virus disease for each region (key) and the approximate known endemic area for Lassa fever (dotted oval line). Sites of the four laboratories included in the Mano River Union Lassa Fever Network (MRU-LFN) are indicated by black stars: Kenema, Sierra Leone; Monrovia, Liberia; Conakry, Guinea; and N'Zérékoré, Guinea. Guéckédou, Guinea, where the Ebola outbreak is thought to have begun, is indicated by a red star. Adapted from the Ebola Response Road Map, December 17, 2014, World Health Organization and Khan et al.^2^

In 2004, the WHO, Tulane University, the Mano River Union country (Guinea, Sierra Leone, and Liberia) governments, and various collaborators and funders established the Mano River Union Lassa Fever Network (MRU-LFN) to assist “in the development of national and regional prevention and control strategies for Lassa fever and other dangerous diseases, including the enhancement of laboratory diagnostic capacity, and training in laboratory diagnosis, clinical management, and infection and environmental control.”^2^ The cornerstone of the project was enhancement of laboratory capacity. Although the initial focus was Kenema, the project was also to enhance capacity in existing laboratories in Monrovia, Liberia; Conakry, Guinea; and most pertinent now, N'Zérékoré, Guinea (Figure 1). The N'Zérékoré laboratory, just a few hours away from the Guéckédou region, where Ebola virus apparently was introduced into humans to start the terrible west African outbreak,^3^ was created during my time working on Lassa fever with the CDC. We eventually discovered that the incidence of Lassa fever and thus, the potential for research was much less in Guinea than in Sierra Leone.^4^ Enthusiasm for the project waned, and 2002 was the last year of the CDC funding. The resources of the MRU-LFN were never sufficient to carry the load in any laboratory other than Kenema, and by 2010, the MRU-LFN had lost much of its steam. Without any real support from the Guinean government or an international benefactor, the N'Zérékoré laboratory's capacity slowly faded. Could Ebola virus, which was identified as the source of the outbreak in laboratories in France and Germany in March of 2014,^3^ 3 months after the first introduction in Guéckédou, have been identified months earlier if the laboratory capacity had existed just down the road in N'Zérékoré?

The MRU-LFN did have some success; excellent laboratory infrastructure was established in Kenema that has proven very valuable in providing Lassa and since the onset of this outbreak, Ebola virus diagnostics. However, the broader goal of the MRU-LFN, to bring the three Mano River Union countries together and give them the tools to mount a coordinated and comprehensive public health response, was sadly never realized—what seems now to be a glaring and tragic missed opportunity.

## Striking the Research-to-Patient Care Balance

**Figure 2.**
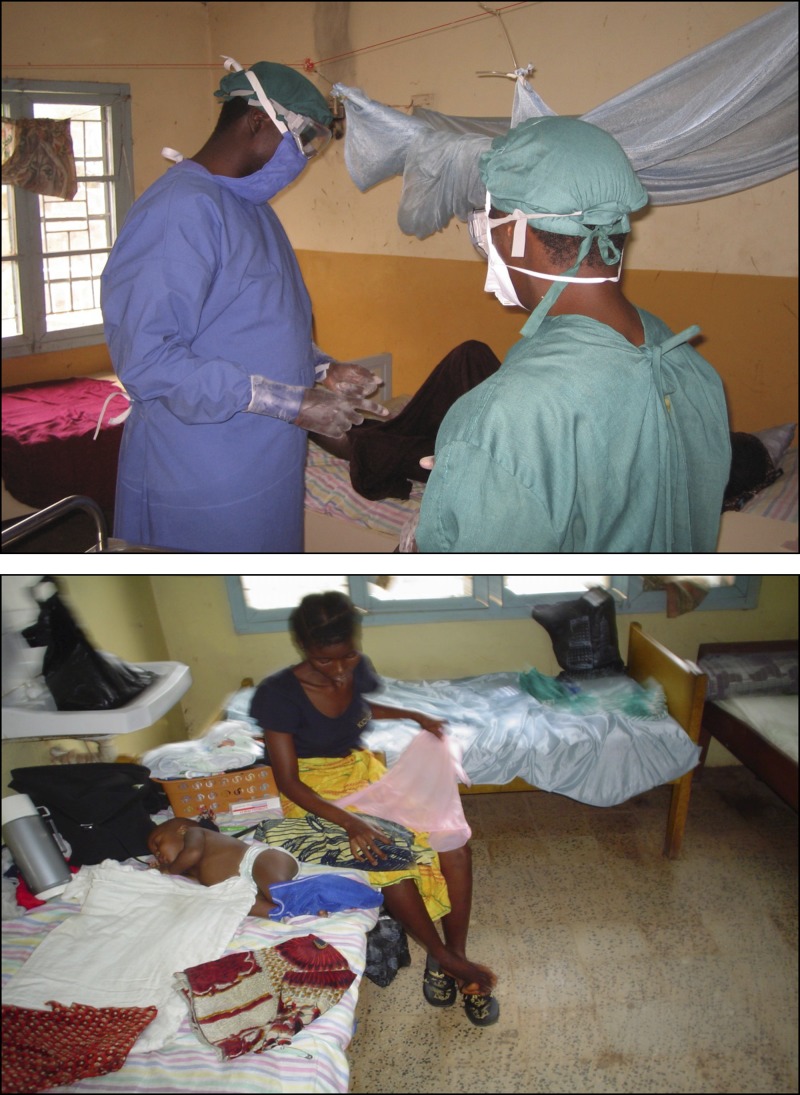
Healthcare workers and patients in the Kenema Government Hospital Lassa ward in 2005. Photos by D.G.B. and A. Wurie.

Starting in 2005, Tulane University and partners began receiving considerable funding from the US National Institutes of Health (NIH) for Lassa fever projects based out of Kenema primarily focused on developing diagnostics and understanding cellular mechanisms and disease pathogenesis. Although these projects led to considerable upgrades in the laboratory infrastructure as well as advances in our understanding of Lassa fever, NIH funding restrictions left little room to support patient care. I always felt bad when comparing the shiny new research and diagnostics laboratory at Kenema Government Hospital with the dilapidated, cramped, and poorly resourced Lassa ward only some 50 m away (Figure 2). Considerations of building a new Lassa ward have been ongoing for over a decade and have even led to ground breaking and initial phases of construction promoted and sponsored at times by the European Union, the WHO, and the US Department of Defense. However, each time, some logistical demon raised its head—contractors had insufficient capital, budgets were frozen, funds were lost, and key personnel changed. The unfinished shell of the new ward in Kenema still sits collecting rain, a testament to good intentions betrayed by the logistical, bureaucratic, and financial complexities of the world of development, although there are plans now to finally finish it off. There were also periodic discussions with Medecins Sans Frontieres-Belgium, which had a malaria project in a neighboring district, about taking over the running of the Kenema Lassa ward, but nothing ever materialized. How much better and safer might Ebola care have been in Kenema if these projects were seen through to completion?

## Training

Through the course of the MRU-LFN program and related projects in Guinea and Sierra Leone, we recognized that we needed to invest in training to create the researchers and public health experts of the region. We chipped away at this by hiring and training technicians and integrating as best we could with the local governments. We created a 2-week masters' level course in public health and tropical medicine held each summer in Kenema in collaboration with the Kenema Eastern Polytechnic University. This offered the innovative feature of pairing Sierra Leonean (at drastically reduced tuition) and American students to expand their mutual academic as well as cultural horizons. A handful of Tulane students would spend summers in Kenema working on various research and public health projects, and we would occasionally find the resources to bring a Sierra Leonean or Guinean collaborator to the United States to visit Tulane and attend a scientific meeting.

However, these were small steps. Real progress could only be made through larger, more comprehensive training programs to provide the doctoral-level foundation required to develop leaders in the health sciences. We wrote some training grant applications here and there submitted to the US Agency for International Development (USAID), the World Bank, and others but had no luck. Major challenges in finding and creating training opportunities included the generally high cost of tuition in the United States, limited English fluency of candidates (especially in francophone Guinea), constant personnel changes in the Ministries of Health and Education of the west African countries, and far-from-intuitive or user-friendly processes for taking the test of English as a foreign language (TOEFL) or graduate record exam (GRE) or identifying training opportunities through US Embassies. I am somewhat ashamed to admit that we have not, to date, succeeded in enrolling one of our African colleagues in a doctoral-level training program.

There were also missed opportunities on the American side; one particularly strong “I told you so” reflection is a 2010 proposal entitled “Training in Patient Management and Clinical Research on Biosafety Level Four Pathogens” submitted to the Western Regional Centers of Excellence (WRCE) in Galveston, TX, one of various centers created by the NIH to support research focused on countering threats from bioterror agents and emerging infectious diseases. Recognizing the dearth of physicians in the United States with experience in the clinical management of viral hemorrhagic fevers, we proposed to rotate US clinicians through the Kenema Lassa fever ward to provide valuable clinical experiences for visiting Americans and hosting Sierra Leoneans alike, while improving the quality of patient care. Although the protocol received very favorable reviews and was recommended for funding by the WRCE, the NIH saw things differently and pulled the plug before we got started.

## A Focus on Patient Care and Field Research on Drugs and Vaccines

This outbreak has brought to the forefront the issue of improving care for the individual patient with Ebola virus disease, including the complex challenge of field-testing drugs and vaccines. However, the matter is not new; in 2006, a group of filovirus experts met in Winnipeg, Canada at a meeting entitled “Marburg and Ebola Hemorrhagic Fever: Feasibility of Prophylaxis and Therapy.” Various manuscripts published in the meeting's wake called for a refocusing on individual patient care,^5^ a byproduct of which would be a better understanding of disease pathogenesis, and charting out a course for clinical testing of drugs and vaccines during outbreaks.^6^ However, there was not much action afterward. Although this meeting and the subsequent publications can hardly be considered a panacea for the present challenges in west Africa, it is hard to believe that we would not be farther down the road toward a solution if more concerted steps had been taken toward the advocated strategies.

## Enhancing Infection Control at Kenema Government Hospital

To date, 24 (89%) of 27 healthcare workers working in the Ebola ward in Kenema have contracted Ebola virus, 19 of them fatally (case fatality rate = 80%), revealing the dismal state of infection prevention and control (IPC) at Kenema Government Hospital. However, realization that IPC needed significant shoring up did not start with the new Ebola outbreak. Numerous healthcare workers contracted Lassa virus in the Lassa ward over the years, including its former chief.^7^ In 2007, a nurse studying for her Masters of Public Health (MPH) degree at Tulane spent a summer in Kenema doing an IPC assessment of the hospital, working with a very capable Sierra Leonean staff nurse. Many IPC shortcomings were identified, about which the Sierra Leonean nurse was enthusiastic and diligent in his desire to remedy. On more than one occasion, he submitted a small budget to address some of the issues, but other research priorities always superseded compounded by the fact that there was no funding line from research grants to address IPC issues. Little ever came of it. How many healthcare worker lives could have been saved if something had?

I know that the issues discussed above are complex, that there are always competing priorities, and that hindsight is 20/20. I know that, in the vast majority of cases, the people involved made the best decisions and did the best job that they could. I know that there are no easy answers neither looking back nor going forward. My comments are not meant to point fingers or assign blame but rather, to provide a degree of honest introspection that can lead to what I also know—that we can do better and that the missed opportunities of the past 10 years must not be missed again.
